# Cholera Researches of Dr. Koch

**Published:** 1884-12-20

**Authors:** 


### CHOLERA-RESEARCHES OF DR. ROBERT KOCH.

Translated srom the Courier des Etats Unis, by S. POLLAK, M. D., St. Louis.

MARSEILLES, July 10.

At a conference of Physicians with Dr. Koch, of the Pharo-Hospital, a careful microscopical examination was made of the microbes, which Dr. Koch brought from India.

He was asked whether he ever found the microbes in the blood. Never, was his answer. He demonstrated that the choleric microbes are half as large as the typhoid microbes, and have, besides, an oblong form. He found them in the most fulminating cases of cholera. In no other epidemic disease did he find the microbe so characteristic as in cholera. The microbe is not sporous. It is a scissiparious product; it lives in an alkaline liquid; is destroyed by acidity, and still more by siccity. Three hours of siccity suffice to kill it in the dejections. Its channels of introduction are the mouth, the digestive organs, and the intestines. If it is in the air it is soon reduced to powder, and enters the lungs, without harm. In the intestines it multiplies rapidly.

When asked by the president of the sanitary commission about prophylactics, he said that the bacilli live in water, and the virulent germ propagates itself. It can be propagated by the washing of vegetables or other aliments. The first measure which should be rigorously prescribed is to use only cooked aliments, in which the microbe cannot exist.

The treatment recommended by him in the beginning is opium, which must be discontinued as soon as the algide state is reached; stimulants may then become necessary, though he could indicate none. As soon as chilliness sets in nothing can be done. He washes his hands in a solution of bichloride of mercury, 1-1000, eats only well-cooked food, and drinks only boiled water.

Before leaving Toulon Dr. Koch addressed the following communication to the Council of Hygiene:

"Cholera propagates itself in assemblages of men; its principle is unexceptionally communicated by direct contact with men, or with their wearing apparel.

"In cholera times a well-regulated life must be led; experience has demonstrated that troubles of digestion favor an attack of cholera. Excesses in eating and drinking, the use of heavy, indigestible food must be avoided, and also anything which may cause diarrhœa.

"A physician should be called in immediately.

"Never absorb anything which comes from a contaminated source; when in doubt whence it came, subject it to a thorough cooking, and especially so milk.

"Water tainted by human detritus is forbidden; avoid the use of doubtful water, of water drawn from shallow wells or a swamp, a pond, or from a creek receiving vitiated water. Consider essentially dangerous water which has been in any manner soiled by choleric dejections. Water which has been used for rinsing vessels, or washing of clothing, should not be poured into a well or into a running stream. It is impossible to get absolutely pure water, the simplest way is, therefore, to have it boiled.

"These observations refer not only to water used for drinking, but to water used in the household, for the choleric germ, once existing in the water can be communicated to all who make use of it, whether in the laundry, scullery, kitchen, or bath. The most important consequences of these remarks are, that it is not sufficient to guard against cholera, to use pure or even boiled water.

"One case of cholera can become the focus of an epidemic, hence cholera patients should be removed from contact with all those not absolutely necessary to take care of them.

"Public assemblages, fairs, fetes, should be avoided. Never eat or drink in a room where the choleric are; their dejections should be collected in vessels containing a phenic solution.

"All objects soiled by the dejections should be cleansed with dry cloths, which have to be burned immediately. Apartments which have been occupied by the choleric should remain vacant for six days.

"Persons who are brought in contact with cholera patients should wash their hands with soap and phenic acid, or solution of bichloride of mercury, 1-1000. Cadavers should be removed immediately, and the funerals should be simplicity itself. The cortege should not enter the mortuary house, and none of the objects belonging to the choleric should be removed without having been previously disinfected.

"Laundresses should not receive the linen coming from a choleric without having undergone thorough disinfection."—*Jour. Am. Med. Association.*

